# A developmental cell-type switch in cortical interneurons leads to a selective defect in cortical oscillations

**DOI:** 10.1038/ncomms6333

**Published:** 2014-10-30

**Authors:** Naoki Takada, Hyun Jae Pi, Vitor H. Sousa, Jack Waters, Gord Fishell, Adam Kepecs, Pavel Osten

**Affiliations:** 1 Cold Spring Harbor Laboratory, Cold Spring Harbor, New York 11724, USA; 2 Smilow Neuroscience Program, the Department of Cell Biology, New York University, New York, New York 10016, USA; 3 Department of Physiology, Feinberg School of Medicine, Northwestern University, Chicago, Illinois 60611, USA

## Abstract

The cellular diversity of interneurons in the neocortex is thought to reflect subtype-specific roles of cortical inhibition. Here we ask whether perturbations to two subtypes—parvalbumin-positive (PV+) and somatostatin-positive (SST+) interneurons—can be compensated for with respect to their contributions to cortical development. We use a genetic cell fate switch to delete both PV+ and SST+ interneurons selectively in cortical layers 2–4 without numerically changing the total interneuron population. This manipulation is compensated for at the level of synaptic currents and receptive fields (RFs) in the somatosensory cortex. By contrast, we identify a deficit in inhibitory synchronization *in vitro* and a large reduction in cortical gamma oscillations *in vivo*. This reveals that, while the roles of inhibition in establishing cortical inhibitory/excitatory balance and RFs can be subserved by multiple interneuron subtypes, gamma oscillations depend on cellular properties that cannot be compensated for—likely, the fast signalling properties of PV+ interneurons.

## Abstract (web summary)

The neocortex contains a large diversity of interneurons but the subtype-specific roles of these cells in establishing excitatory and inhibitory circuits are not well understood. Here the authors genetically delete parvalbumin- and somatostatin-positive interneurons during mouse development and study the functional effects in postnatal cortical circuits.

## Graphical abstract

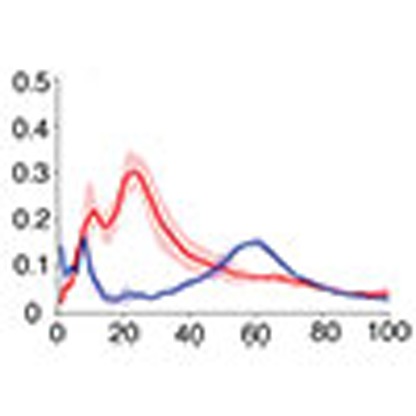

GABAergic interneurons have traditionally been classified into distinct subtypes based on their cellular properties, including firing properties, morphological features and expression of proteins linked to synaptic functions, such as the calcium-binding proteins parvalbumin (PV) and calretinin (CR) or co-neurotransmitters somatostatin (SST) and vasoactive intestinal peptide (VIP)^1^. The extraordinary diversity of interneuron subtypes in the mammalian neocortex suggests that inhibition plays particularly important and complex roles in controlling the development and adult functions of cortical excitatory circuits.

Genetic lineage-tracing studies have revealed detailed information about the embryonic origins of the main cortical interneuron subtypes, as well as their migration and final distribution across cortical layers. The most abundant cortical subtypes are the PV+ and SST+ interneurons, which arise from the medial ganglionic eminence (MGE) and account for nearly 70% of cortical inhibitory cells^2^,^3^,^4^. The PV+ interneurons are characterized as fast spiking and include basket cells populating cortical layers 2–6 and chandelier cells found in layers 2 and 5/6, whereas the SST+ interneurons comprise the Martinotti cells and distribute to layers 2/3 and 5. The remaining ~30% of cortical interneurons arise from the caudal ganglionic eminence and can be broadly classified as bipolar VIP-positive (VIP+), the CR-positive (CR+) interneurons and the reelin-expressing neurogliaform cells, all populating mainly superficial cortical layers 2/3 (ref. 4). Remarkably, a single transcription factor, called *Nkx2-1*, acts as a ‘master switch’ in promoting the cell fate of the MGE-derived interneurons: Nkx2.1 is expressed only in the MGE and its genetic deletion leads to a cell fate switch of the PV+ and SST+ subtypes into caudal ganglionic eminence-like VIP+, CR+ and reelin+ subtypes as defined by the expression of the respective marker proteins, firing properties and axonal arborizations^5^,^6^.

In contrast to the detailed knowledge about the embryonic origins, we know much less about subtype-specific roles of cortical interneurons during postnatal development, when excitatory and inhibitory circuits are established and shaped by sensory experience. On the basis of the studies of a knockout mouse model lacking the glutamic acid decarboxylase 65 (GAD65) protein, one of the two GABA-synthesizing enzymes in the brain, the onset of normal receptive field (RF) development is proposed to depend on the maturation of GABAergic circuits that is needed to reach a necessary threshold level of inhibition and establish normal excitation/inhibition (E/I) balance^7^,^8^,^9^. The developmental increase in inhibition has been proposed to be mediated by the PV+ interneurons, because their maturation in the cortex approximately parallels RF development^7^. However, it is not known whether specific cellular properties of PV+ interneurons are required for normal cortical development, including establishing the E/I balance and sensory RFs, or whether the overall increase in inhibition (independent of subtype-specific roles) is the critical developmental factor.

In the adult cortex, subtype-specific roles of inhibition have recently begun to be elucidated by optogenetic manipulations. The PV+ interneurons are proposed to drive gamma (30–80 Hz) oscillations, thought to be a substrate for neuronal coordination underlying cortical processing. The fast signalling properties of PV+ interneurons, such as the ability to fire short-duration action potentials, rapid and highly synchronized GABA release and short inhibitory postsynaptic current (IPSC) decay time constant, have been proposed as the underlying cellular mechanisms that enable this cell type to organize the fast network oscillations^10^,^11^,^12^,^13^,^14^. This can occur either via the activation of reciprocally connected inhibitory circuits, termed interneuron gamma, or via an excitatory-inhibitory loop, termed the pyramidal-interneuron gamma^10^,^15^,^16^,^17^. In support of the PING model in the cortex, optogenetic activation of either the PV+ interneurons or layer 2/3 pyramidal neurons was shown to be sufficient to initiate cortical gamma activity^18^,^19^,^20^. Other optogenetic studies showed that the activity of PV+ interneurons can control the size as well as the gain of sensory RFs^21^,^22^,^23^, while the activity of SST+ interneurons can regulate the RF size in the visual cortex^24^,^25^. These studies suggest that the adult requirements for modes of cortical inhibition are allocated in a subtype-specific manner. They do not, however, address the importance of interneuron subtype diversity during development, including the question whether the PV+ and SST+ subtypes are necessary for the proposed roles or whether some inhibitory circuit functions may be compensated for by other interneurons.

Here we study the developmental cell type-specific roles of cortical inhibition by genetically deleting the PV+ and SST+ interneurons in the upper layers of the neocortex. This manipulation enabled us to test whether the innervation by the two interneuron subtypes is necessary for normal development of pyramidal neuron cellular (intrinsic) and synaptic properties, cortical E/I balance, sensory RFs and network synchronization.

## Results

We used a genetic approach based on a conditional deletion of the MGE-specific Nkx2.1 in a loss-of-function (*Nkx2-1*^*LOF*^) mouse model^6^, which allowed us to test the roles of the PV+ and SST+ interneurons during development and in the young adult. We timed the Nkx2-1 deletion at embryonic day (E) 12.5 (*Nkx2-1*^*E12.5LOF*^), which restricts the cell fate switch to the cortical layers 1–4 and causes the substitution of the PV+/SST+ interneurons by the VIP+, CR+ and neurogliaform subtypes without affecting the total interneuron cell count^5^,^6^. Given that the *Nkx2-1*^*E12.5LOF*^ mice have normal lifespans and no seizures^6^, we hypothesized that the loss of PV+ and SST+ interneurons may be compensated for at the level of gross cortical development and, therefore, that these mice may allow us to directly test whether these interneuron subtypes are necessary for the development of cortical E/I balance and sensory RFs, as well as the generation of gamma cortical oscillations.

### Characterization of the Nkx2-1 deletion *in vitro*

**Figure 1:**
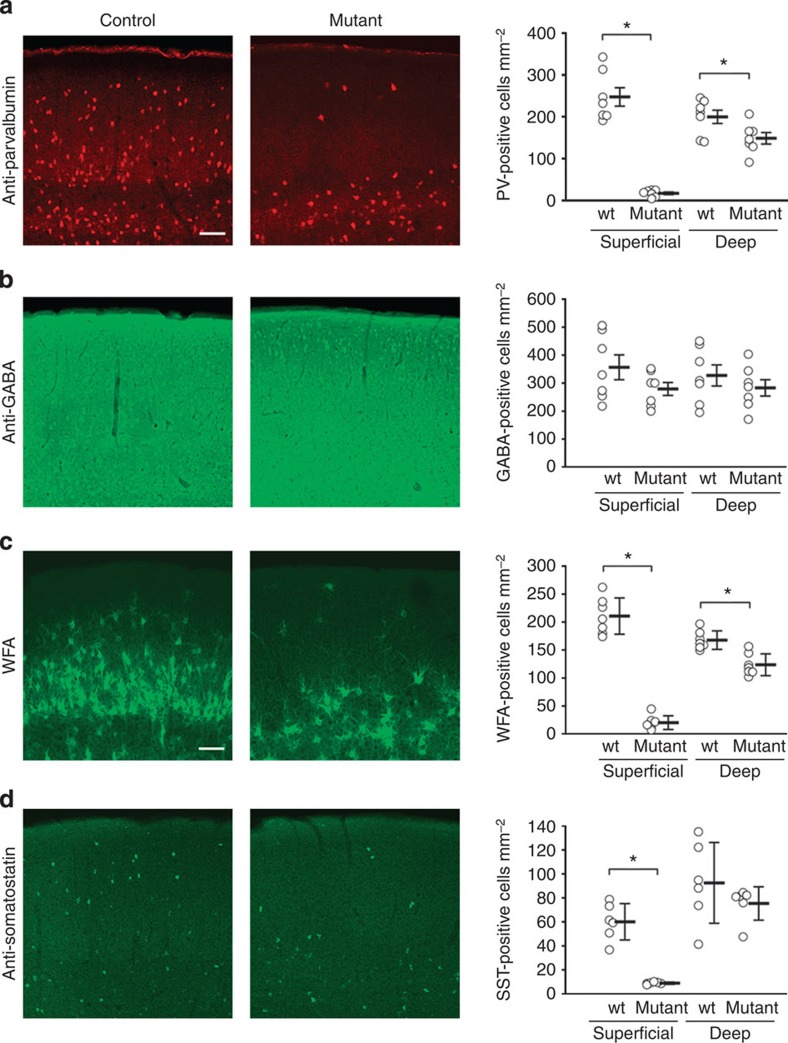
Quantification of the *Nkx2-1*^*E12.5LOF*^ cell fate switch. (**a**,**b**) Double fluorescent immunostaining against PV (**a**) (*n*=7 wt and 7 mutant) and GABA (**b**; *n*=7 wt and 7 mutant). (**c**) Fluorescent immunostaining against WFA, a marker of PV-specific perineuronal nets^55^ (*n*=7 wt and 7 mutant). (**d**) Anti-SST immunostaining (*n*=6 wt and 6 mutant). Asterisks indicate statistical significance *P*<0.05 (two-tailed *t*-test). Error bars are s.e.m. Scale bar, 100 μm. Each staining is an example of two repeated experiments.

In the first set of experiments, we confirmed by immunohistochemistry that the E12.5 deletion of Nkx2-1 reliably induces the cell fate switch as intended^6^, demonstrating ~93% reduction in PV+ and ~85% reduction in SST+ without any changes in the total interneuron count as revealed by anti-GABA immunostaining in the superficial layers of the somatosensory cortex (Fig. 1; Supplementary Table 1).

We then considered the consequences of this manipulation on the development of excitatory layer 2/3 pyramidal neurons. First, we tested whether the development of biophysical and cellular properties of pyramidal neurons is affected by the altered interneuron composition. If so, such changes would need to be taken into account when interpreting more complex cortical functions in later experiments. Whole-cell recordings in acute brain slices from control and *Nkx2-1*^*E12.5LOF*^ mice revealed no differences in intrinsic membrane properties, including input resistance, rectification index and membrane time constant (Supplementary Tables 2 and 3). Several other cellular properties, including action potential properties, resting membrane potential and excitability, were altered during the second postnatal week (P11–13), but returned mostly to normal by the end of the third week (P20–22) (Supplementary Tables 2 and 3; Supplementary Figs 1 and 2). These data show that the *Nkx2-1*^*E12.5LOF*^ manipulation induces transient changes in the development of cellular properties of cortical pyramidal neurons, which are largely compensated for by the end of the third postnatal week marking the end of the critical period of sensory development in the somatosensory barrel cortex^26^,^27^.

**Figure 2:**
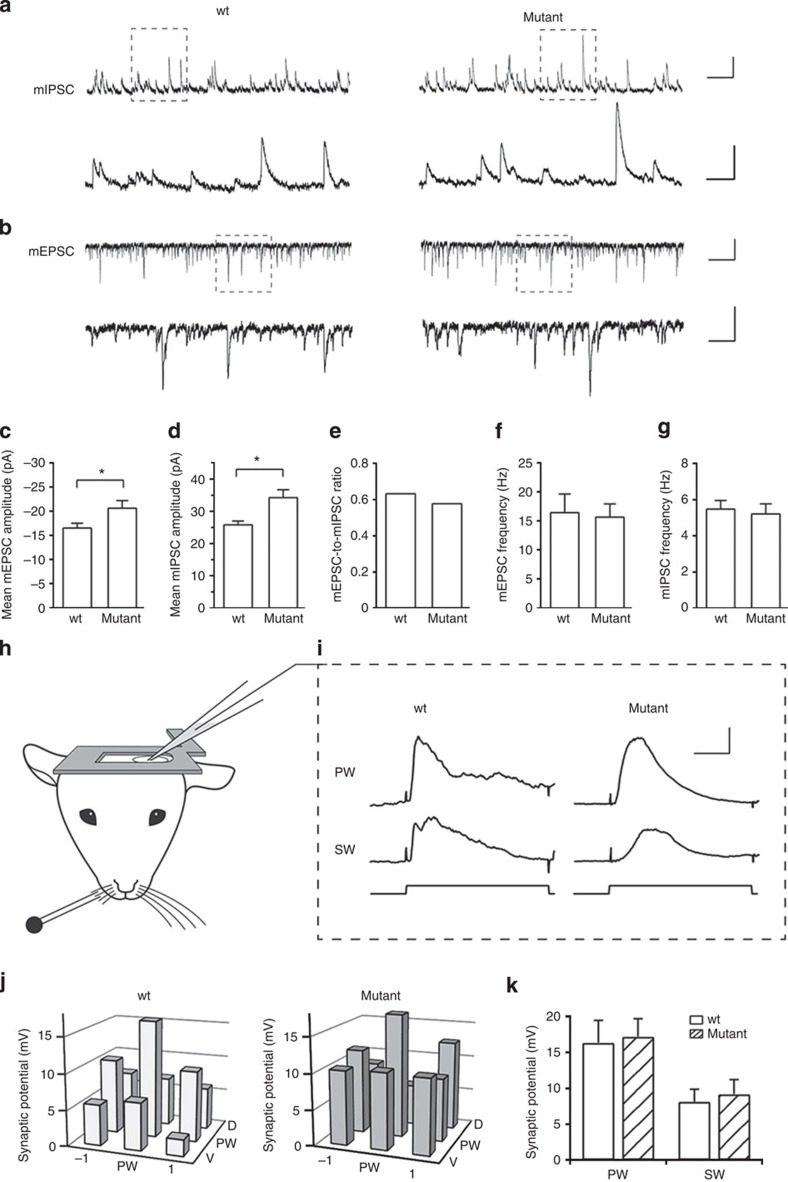
Synaptic E/I balance and sensory RFs of layer 2/3 pyramidal neurons in the *Nkx2-1*^*E12.5LOF*^ layer 2/3 somatosensory cortex. (**a**) Sample mIPSC (top) and (**b**) mEPSC (bottom) traces from layer 2/3 pyramidal neurons in the somatosensory cortex in control (wild type, wt) and *Nkx2-1*^*E12.5LOF*^ (mutant) acute brain slices. Scale bars, 500 ms and 30 pA ((**a**) top) and 100 ms and 50 pA (bottom); and 50 pA and 500 ms ((**b**) top) and 50 pA and 100 ms (bottom). (**c**–**e**) Analysis of mEPSC (*n*=11 wt; *n*=12 mutant) and mIPSC (wt *n*=11; mutant *n*=16) mean amplitudes. Error bars are s.e.m. (**f**,**g**) Mean frequency of mEPSCs (**e**) and mIPSCs (**f**). Error bars are s.e.m. Asterisks indicate statistical significance *P*<0.05 (two-tailed *t*-test). (**h**) A schematic representation of the recording setup in an anaesthetized mouse, showing a craniotomy window with a patch pipette at a site contralateral to piezo-based deflection of the facial whiskers. (**i**) Sensory responses evoked by deflection of principal and surrounding whiskers (PW and SW1, respectively) in layer 2/3 pyramidal neurons. The traces are averages of about 20 responses. The duration of the whisker deflection is indicated schematically below the traces. Scale bar, 50 ms and 5 mV. (**j**) RF maps of control (*n*=5) and *Nkx2-1*^*E12.5LOF*^ (*n*=8) mice shown centred on the principal whisker; each bar indicates the average amplitude of postsynaptic potentials evoked by the deflection of corresponding whiskers. (**k**) Mean amplitude of whisker-evoked postsynaptic potentials in primary whisker (PW) or primary SW1 stimulations in control and *Nkx2-1*^*E12.5LOF*^ mice (mV; mean±s.e.m.): control PW=16.36±3.10, mutant PW=17.21±2.47, *P*=0.83; control SW=8.16±1.70, mutant SW=9.19±2.03, *P*=0.72 (two-tailed *t*-test).

Next, we determined whether normal balance of synaptic excitation and inhibition is preserved in the *Nkx2-1*^*E12.5LOF*^ somatosensory cortex, using whole-cell recordings in brain slices from 3- to 4-week-old animals. First, recordings of spontaneous miniature excitatory postsynaptic currents (EPSCs) and IPSCs (mEPSCs and mIPSCs, respectively) in layer 2/3 pyramidal neurons revealed no changes in mEPSC frequencies and comparably increased mEPSC and mIPSC amplitudes, by ~24 and ~32%, respectively, resulting in no change in the mEPSC/mIPSC amplitude ratio in *Nkx2-1*^*E12.5LOF*^ compared with control slices (Fig. 2a–g; Supplementary Table 4). Second, we also tested evoked EPSCs and IPSCs by incubating the brain slices in a bath solution with higher potassium and lower magnesium concentration^28^. As shown in Supplementary Fig. 3, this analysis also did not detect any differences between the two conditions (Supplementary Table 4). These data thus indicate that the cortical E/I balance is normal in the *Nkx2-1*^*E12.5LOF*^ mice at 4 weeks of age. The transient changes in cellular properties and the comparable increase in mEPSC and mIPSC amplitudes suggest that some forms of homeostatic synaptic plasticity^29^ likely contribute to the normalization of synaptic currents and E/I balance in the *Nkx2-1*^*E12.5LOF*^ cortex.

### Nkx2-1 deletion does not affect RF properties

Having demonstrated largely normal properties of excitatory pyramidal neurons and normal E/I balance in the *Nkx2-1*^*E12.5LOF*^ cortex, we turned our attention to the study of sensory RF development, by mapping sensory responses in the somatosensory barrel cortex representing inputs from the rodent facial vibrissae (whiskers). The critical period in the barrel cortex occurs during the second to third postnatal week, when the sensory responses of layer 2/3 neurons increase in strength and their RFs are sensitive to sensory deprivation^26^,^27^. Mature RFs of layer 2/3 pyramidal neurons in the barrel cortex have the strongest response to the deflection of the principal whisker and weaker responses to the deflections of the adjacent surround whiskers^30^. Whole-cell recordings *in vivo* in anaesthetized mice (~4 weeks old) revealed normal layer 2/3 RF properties in the *Nkx2-1*^*E12.5LOF*^ barrel cortex (Fig. 2h–k). The amplitude of whisker-evoked subthreshold postsynaptic potentials was (mV): principal whisker: 16.4±3.1 and 16.3±2.4 (*P*=0.996; Student’s *t*-test); and surround whisker: 8.2±1.7 and 8.7±1.9 (*P*=0.843; mean±s.e.m.), in control and *Nkx2-1*^*E12.5LOF*^ recordings, respectively. In addition, no differences were observed in the postsynaptic potential kinetics (Supplementary Table 5) or in the number of sensory-evoked action potentials (spikes per trial): 0.064±0.032 and 0.067±0.040 (*P*=0.954), control and *Nkx2-1*^*E12.5LOF*^ barrel cortex, respectively. On the basis of these data, we conclude that the cell fate switch of the PV+ and SST+ interneurons is compensated for at the level of somatosensory RF development in the *Nkx2-1*^*E12.5LOF*^ barrel cortex.

### Nkx2-1 deletion prevents fast cortical oscillations

**Figure 3:**
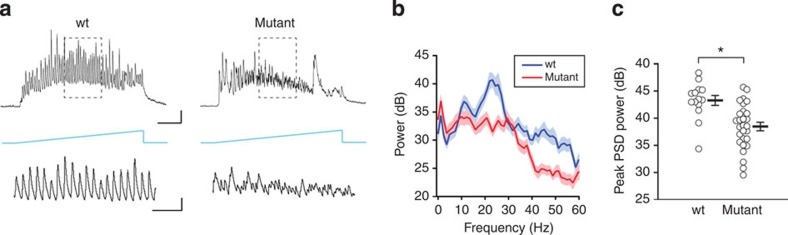
ChR2-induced oscillations of inhibitory currents. (**a**) Ramp of blue light was used to drive excitatory activity in ChR2-YFP-expressing pyramidal neurons and the evoked IPSC oscillations were recorded in layer 2/3 neurons. Scale bar, 0.5 s and 500 pA (top); 0.1 s and 500 pA (bottom). The bottom traces show expanded sections of the traces in the dashed boxes. (**b**) The averaged PSD of IPSC oscillations. s.e.m. is represented by the area above and below the average traces. (**c**) The summary of the power at the peak PSD frequency. The peak power was significantly smaller in the mutant: control: 43.256±0.917; mutant: 38.477±0.781; *t*-test: *P*=0.0004 (*n*=14 wt and 28 mutant). Asterisk indicates a statistical significance from control values.

Next, we turned our attention to the study of cortical circuit dynamics, first using an optogenetic model of cortical oscillations driven by activation of layer 2/3 pyramidal neurons^20^. Channelrhodopsin-2 (ChR2) was targeted to layer 2/3 pyramidal neurons by stereotaxic injections of an adeno-associated virus expressing yellow fluorescent protein (YFP)-tagged ChR2 (ChR2-YFP) under the control of the alpha-CaMKII promoter^18^,^31^. Driving ChR2-expressing pyramidal neurons with a ramp of blue light (2 s of increasing intensity) was sufficient to induce a robust synchronization in control brain slices, as manifested by an increase in IPSC power in the 20–30 Hz frequency band in whole-cell recordings from layer 2/3 pyramidal neurons (Fig. 3). In contrast, the same manipulation evoked variable activity with a broadly reduced peak power in layer 2/3 neurons in *Nkx2-1*^*E12.5LOF*^ brain slices (Fig. 3), indicating an impaired capacity of local inhibitory circuits to synchronize at higher frequencies. This suggests that the inhibitory circuits in the *Nkx2-1*^*E12.5LOF*^ cortex may not be able to sustain fast oscillations *in vivo* in behaving mice.

**Figure 4:**
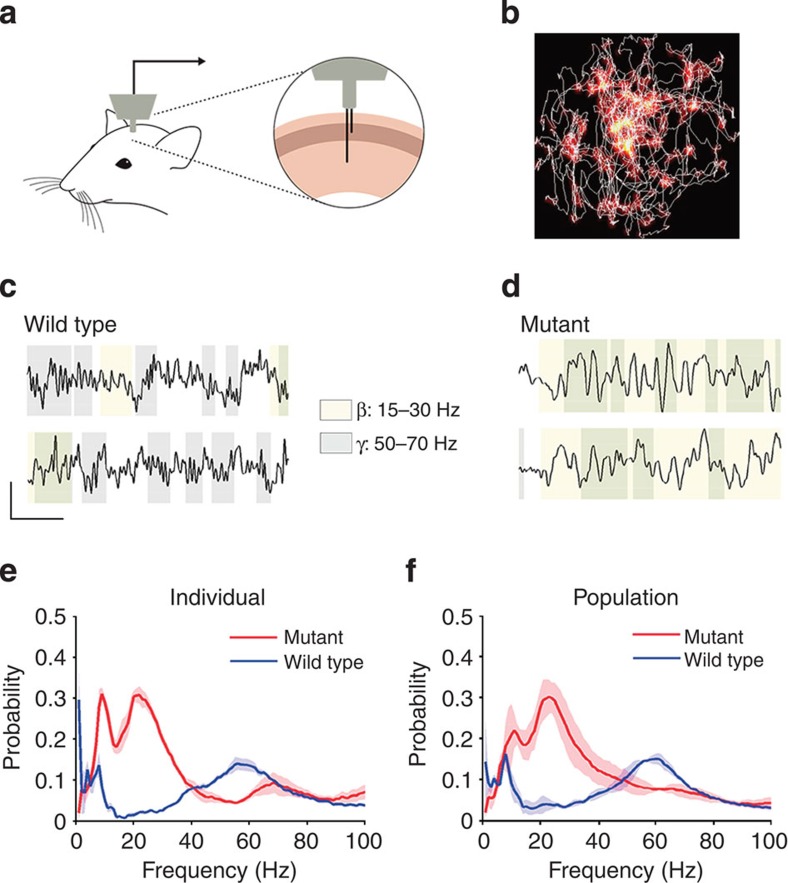
LFP recordings in the barrel cortex in awake free-moving mice. (**a**) Schematic illustration of the setup of LFP recordings. One microdrive electrode was inserted in the upper layers and one in the deep layers in the barrel cortex. (**b**) LFPs were obtained in a novelty-induced exploration condition, where the animals were actively whisking and exploring around the novel objects in the arena. Heat map shows the occupancy of the animal in the novel arena during LFP recordings and the trace indicates a segment of track that the animal navigated on. (**c**–**d**) Sample traces of LFPs (left traces, control; right traces, mutant; upper traces, recordings from the upper layers; lower traces, recordings from the deep layers). The beta frequency component is highlighted in yellow while the gamma component is in gray. (**e**) The probability distribution of oscillatory frequency component computed by the better oscillation detection method (BOSC) in a pair of the control (blue line) and mutant (red line). (**f**) The summary data of BOSC analysis shown in **d** (*n*=3 pairs of mutant and wild-type mice). The high-frequency gamma component is diminished in the mutant and instead the beta component becomes dominant.

In the final sets of experiments, we addressed the question whether fast cortical oscillations persist in the *Nkx2-1*^*E12.5LOF*^ mice during behaviour. Local field potentials (ΔLFP in bipolar configuration between supragranular and infragranular layers) were recorded in the barrel cortex in control and *Nkx2-1*^*E12.5LOF*^ mice during a novelty-induced exploration paradigm. During exploration, behavioural measures were not different between control and *Nkx2-1*^*E12.5LOF*^ animals (mean±s.e.m.): occupancy of a novel object (novel object visit/total time): 0.07±0.018 and 0.07±0.042 (*P*=0.917); speed of movement (m s^−1^): 0.046±0.0055 and 0.037±0.0014 (*P*=0.146); and distance covered (m): 8.26±0.741 and 6.92±0.151 (*P*=0.081), respectively. However, we observed a dramatic difference between control and *Nkx2-1*^*E12.5LOF*^ mice in terms of oscillations (Fig. 4c,d). To focus on the rhythmic components of the LFP (instead of total spectral power that includes the non-rhythmic 1/f background), we used the better oscillation detection (BOSC) spectral analysis method. This technique detects oscillatory episodes based on power and duration threshold of the wavelet-filtered signal^32^,^33^. The probability of oscillatory episodes was highest around the gamma frequency range in control mice, whereas in the *Nkx2-1*^*E12.5LOF*^ mice the gamma frequency component was nearly absent and the peak frequency was shifted to the lower beta frequency range (Fig. 4). These results thus demonstrate that the PV+/SST+ cell-type switch indeed interferes with the capacity of cortical circuits to generate gamma oscillations in the somatosensory barrel cortex during behaviour, as predicted based on our optogenetic experiments in brain slices.

## Discussion

Our study answered three questions regarding the role of GABAergic inhibition during development and in the young adult cortex. First, we showed that genetic deletion of two major interneuron cell types, the PV+ and SST+ interneurons, can be largely compensated for at the level of cortical E/I balance development when the total interneuron cell count remains unchanged. This is in contrast to the PV+/SST cell fate switch induced by the Nkx2.1 deletion at E10.5, just 2 days earlier than the timing used here, which induces a significant reduction in the number of cortical interneurons and leads to pronounced spontaneous seizures^6^. We have also observed transient changes in the *Nkx2-1*^*E12.5LOF*^ pyramidal neuron cellular properties during the second postnatal week, which were largely compensated for by the end of the third week. The second-to-third week period marks the critical period of RF development in the somatosensory barrel cortex^26^,^27^. This suggests that some forms of homeostatic plasticity, known to play important roles during postnatal development^29^, are activated and contribute to the establishment of E/I balance.

Second, the finding of normal sensory-evoked responses in the *Nkx2-1*^*E12.5LOF*^ barrel cortex suggests that the PV+/SST+ cell types can be also compensated for during somatosensory RF development. The role of inhibition in RF development was examined first in the visual cortex in the GAD2 knockout mice, in which the lack of the GAD65 isoform was shown to lead to a reduction in fast inhibitory transmission, enhanced sensory-evoked responses and a deficit in monocular deprivation-induced plasticity^9^. PV+ interneurons are likely to play a critical role in mediating normal RF development and plasticity, because their maturation occurs approximately in parallel to critical periods of RF development in sensory cortices and their responses are modulated by monocular deprivation^7^,^28^,^34^,^35^. Thus, our data do not exclude a role of PV+ interneurons in this process, but support a model in which the overall level of inhibition mediated by PV+ and possibly other interneuron subtypes and E/I balance^7^, rather than specific cellular properties of the PV+ interneurons, is necessary for normal RF development. In addition, although our data show that the PV+ and SST+ interneurons are not necessary for normal RF development in the barrel cortex, this finding should not be interpreted to mean that these cell types do not regulate RFs in the normal brain. For example, optogenetic studies showed that PV+ interneurons can regulate both the size and the gain of sensory RFs^21^,^22^, while the activity of SST+ interneurons was proposed to regulate the RF size by the suppressive surround mechanism in the adult visual cortex^24^,^25^.

Third, in contrast to the overall normal synaptic currents and sensory-evoked responses, the PV+/SST+ cell fate switch induced large changes at the level of cortical network activity, including a loss of cortical gamma frequency and an augmentation of lower-frequency oscillations in the *Nkx2-1*^*E12.5LOF*^ mice. While several studies have demonstrated that cortical gamma activity can be externally manipulated by multiple means, including optogenetic activation of pyramidal neurons and PV+ interneurons, glutamate AMPA/kainate and metabotropic receptor-mediated excitation and cholinergic neuromodulation^18^,^19^,^20^,^36^,^37^,^38^,^39^,^40^,^41^,^42^, our study demonstrates that the MGE-derived interneuron subtypes are necessary for the generation of cortical gamma rhythms.

Our study does not directly address the question of which interneuronal cellular properties are necessary for the generation of gamma oscillations. On the basis of modelling studies, the lack of fast signalling properties of the PV+ interneurons, including synaptic kinetics, rapid action potentials and high intrinsic resonance frequency, is likely to play a critical role in the observed oscillatory phenotype^13^,^14^,^43^. In addition, the SST+ interneurons, which innervate distal dendrites of cortical pyramidal neurons, have also been shown to synchronize even though at lower (<30 Hz) frequencies^44^. Therefore, the genetic deletion of SST+ interneurons may also contribute to the oscillatory phenotype observed in the *Nkx2-1*^*E12.5LOF*^ cortex.

In summary, our study describes distinct developmental roles of cortical inhibition—cell type-independent regulation of E/I balance and sensory RFs and cell type-dependent regulation of fast cortical oscillations. To our knowledge, this is the first demonstration of a selective deficit in neuronal network synchronization, in the absence of other synaptic network changes. Since the E12.5 *Nkx2.1-1* deletion does not affect interneuron composition in the hippocampus or elsewhere in the brain^6^, our study opens the door to future investigations using the *Nkx2-1*^*E12.5LOF*^ mouse model to probe the roles of cortical gamma oscillations in cognitive behaviours.

## Methods

All data are presented as mean±s.e.m. Statistical significance was tested using the Student *t*-test or Mann–Whitney *U*-test, as stated in the Figure legend texts (significance level <0.05). Animal procedures were approved by the Cold Spring Harbor Laboratory Animal Care and Use Committee. All animals were housed under constant temperature and light conditions (12 h cycle lights ON: 0600, lights OFF: 1800) and given food and water *ad libitum*.

### Generation of Nkx2-1^E12.5LOF^ mice

Triple-heterozygote male mice (*Nkx2-1*^*+/−*^ (ref. 45); *Olig2*^*CreER/+*^ (ref. 46); *Z/EG*^*+/−*^ (ref. 47)) were intercrossed with Nkx2-1 conditional homozygote females (*Nkx2-1*^*C/C*^) (ref. 48) to generate experimental control (Nkx2-1^C/+^; Olig2^CreER/+^; Z/EG^+/−^ or Z/EG^−/−^) and mutant (Nkx2-1^C/−^; Olig2^CreER/+^; Z/EG^+/−^ or Z/EG^−/−^) mice. Tamoxifen (4 mg, Sigma) was administered to pregnant mice at E12.5 to induce the Cre recombinase, leading to a cell fate switch of superficial cortical interneurons derived from the MGE^6^.

### Immunocytochemistry

The mice were killed by transcardial perfusion with 4% paraformaldehyde and the brains were dissected and sectioned coronally at 50 μm. Sections comprising the somatosensory cortex were incubated in a blocking solution containing 5% donkey serum and 0.2% Triton X-100 in phosphate buffer for 1 h, followed by the incubation with the blocking solution containing mouse anti-parvalbumin (Sigma, 1:1,000) or rat anti-somatostatin (Millipore, 1:200) antibodies or fluorescein-labelled Wisteria floribunda lectin (Vector Laboratories, 1:500), in combinations with rabbit anti-GABA (Sigma, 1:500) or guinea pig anti-type 2 vesicular glutamate transporter (VGluT2, Millipore, 1:1,000) antibodies overnight at 4 °C. Primary antibodies were fluorescently labelled by incubation with appropriate secondary antibodies conjugated with Alexa Fluor-405, -488 and -594 (1:200, Invitrogen). Fluorescence images were taken using LSM710 confocal laser-scanning microscope (Zeiss). VGluT2 staining was used to visualize barrel structures of the somatosensory barrel cortex, based on the dense labelling of axon terminals from thalamocortical projections^49^. The specific interneuron cell types were counted manually using FIJI image processing software.

### *In vitro* whole-cell patch-clamp recordings

Acute brain slices including the barrel cortex were made by cutting the brain at 45° angle between the horizontal and sagittal plane^50^ in an ice-cold artificial cerebrospinal fluid (ACSF) containing (in mM): 125 NaCl, 2.5 KCl, 1 CaCl_2_, 4 MgCl_2_, 25 NaHCO_3_, 1.25 NaH_2_PO_4_, 25 glucose, 1 kynurenic acid, equilibrated with a mixture of 95% O_2_ and 5% CO_2_. The thickness of slices is 300 μm and the age of animals is postnatal day 20–25 (P20–26), unless otherwise noted. The holding chamber was maintained at 29±1 °C for about 30 min and then kept at room temperature.

Membrane currents and potentials were recorded by using whole-cell patch-clamp techniques (MultiClamp 700B patch-clamp amplifier, Molecular Devices) at 29 °C. The ACSF contained (in mM): 125 NaCl, 2.5 KCl, 2 CaCl_2_, 1 MgCl_2_, 25 NaHCO_3_, 1.25 NaH_2_PO_4_, 25 glucose (pH 7.4), unless otherwise noted. The slices were observed under an upright microscope (BX50WI, Olympus) equipped with a × 40 water immersion objective and IR-DIC optics via a CCD (charge-coupled device) camera (ORCA, Hamamatsu). Glass micropipettes fabricated from borosilicate glass capillaries were filled with the intracellular solution (tip resistance: 4–7 MΩ) containing (in mM): 135 K-gluconate, 4 KCl, 10 HEPES, 4 MgATP, 0.3 Na_3_GTP, 10 phosphocreatine-2Na (pH 7.35) for current-clamp recordings and 100 CsMeSO_3_, 5 CsCl, 10 HEPES, 10 BAPTA-4Cs, 4 MgATP, 0.3 Na_3_GTP, 10 phosphocreatine-2Na, 4 QX-314, 0.3% biocytin (pH 7.35) for voltage-clamp recordings. Series resistance was typically <20 MΩ. Signals were filtered at 1–4 kHz, digitized at 5–20 kHz (USB-6259, National Instruments) and acquired by a custom-made program written in LabVIEW (National Instruments). Miniature EPSCs were recorded at −70 mV in ACSF containing 1 μM tetrodotoxin (TTX), 10 μM SR-95531 (gabazine) and 5 μM (RS)-CPP, whereas miniature IPSCs were recorded at +15 mV in the presence of 1 μM TTX, 5 μM 2,3-dioxo-6-nitro-1,2,3,4-tetrahydrobenzo[f]quinoxaline-7-sulfonamide (NBQX) and 5 μM (RS)-CPP (all drugs were purchased from Tocris). Miniature synaptic events were detected based on the amplitude threshold of 5 pA and fitting of the events with an alpha function of 1 ms rise and 2 ms decay time constants for miniature EPSCs and 2 ms rise and 20 ms decay time constants for miniature IPSCs, followed by visual inspection to exclude inappropriate ones such as overlapped events or events on noisy baseline. Spontaneous synaptic activities without TTX were observed in slightly excitable ACSF in which 2.5 mM KCl and 1 mM MgCl_2_ were replaced with 4 mM KCl and 0.5 mM MgCl_2_, respectively^28^. EPSC and IPSC components were recorded at −60 and +15 mV, respectively, which are close to the reversal potential of the other synaptic component. Because of high occurrences of events, the spontaneous activities were evaluated by calculating the area or charge of the responses. All data analyses were performed by using Igor Pro (WaveMetrics).

### *In vivo* whole-cell patch-clamp recordings

Animals (P29–93) were anaesthetized with urethane (1.5–2 g per kg body weight, intraperitoneal). After incision of scalp, a metal plate was attached to the skull with dental cement to fix a head and make a chamber, and a craniotomy of about 2 mm diameter was made over the barrel cortical region. The dura mater was carefully removed with a needle while the chamber was superfused with ACSF. The exposed surface was then covered with 1.5% agarose. *In vivo* whole-cell patch-clamp was obtained by a ‘blind’ technique^51^. The patch pipette and the internal solution were the same as those used in slice experiments. Initially, LFP recording was performed in layer 2/3 to estimate the primary whisker of the target recording site by stimulating whiskers of the opposite side randomly. Then, a micropipette electrode that has a positive pressure of 30–40 mbar was inserted into the target region while the current response is continuously monitored in a voltage-clamp mode. Positive pressure was released when the resistance suddenly increased, indicating that the tip of the pipette may have been pushed against a cell membrane. Gentle suction was then applied to achieve a GΩ seal if needed. The whole-cell recording configuration was established by voltage clamp, with series resistances between 10 and 75 MΩ^51^. For current-clamp recordings, bridge balance was adjusted manually to eliminate voltage errors arising from series resistance. Sensory stimulations were done as described^52^, by delivering mechanical stimuli (9.5° deflection angle) to each whisker of the opposite side by a capillary attached to a piezoelectric apparatus (Piezo Systems) for 200 ms at a frequency of about 1 Hz. Recordings were obtained with a MultiClamp 700B patch-clamp amplifier, filtered at 10 kHz and digitized at 10–20 kHz using a similar system to that of slice experiments. After recordings, animals were perfused transcardially with 4% paraformaldehyde in phosphate buffer.

### LFP recordings in awake animals

All experiments were performed with the experimenter blind to strain of mice. Adult (over 2 months old) Nkx2-1 mutants and their control litter mates were implanted with custom-built microdrives in the left barrel cortex (1.0 mm posterior to bregma and 3.0 mm lateral to midline) using stereotaxis as described previously^53^. One electrode was placed in the superficial layer (100 μm deep from the pia mater) and the other was in the deeper layer (500–600 μm deep). After 10 days recovery from the surgery, LFPs were obtained using the Cheetah system (Neuralynx) in a novelty-induced exploration condition, where the animals were actively whisking and moving around the novel objects in the arena. Spectral analysis was performed with multitaper technique in Chronux software^54^ and the better oscillation detection method (BOSC)^33^ using Matlab (Mathworks).

### Photostimulation by ChR2

ChR2 fused to YFP was selectively transfected in L2/3 pyramidal neurons in the somatosensory cortex by a stereotaxic injection of adeno-associated viral vectors based on the α-CaMKII promoter. The viral solution of about 90 nl was injected into the barrel cortical region at P13 or 14 (posterior 1.2 mm and lateral 3.0 mm relative to bregma and depth 0.25 mm from the dura mater) and the brain slices were prepared 7–12 days after the injection. After whole-cell recording was established from a cell located in the centre of ChR2-expressing area, ChR2 was activated by illumination of a blue light at around 470 nm through the objective using an LED (LEDC5, Thorlabs), which is attached to the microscope and controlled by the patch-clamp software. The illumination of ramped intensity for 3 s could effectively induce the oscillatory synaptic activities in control neurons. The IPSCs were measured from the trace between 2 and 3 s with respect to the onset of light stimulation. The IPSC decay time constants were measured by fitting with a single exponential function. The degree of rhythmicity was evaluated with a highest frequency component and its intensity of the PSD.

## Author contribution

N.T. carried out all experiments, except *in vivo* LFP recordings that were done by H.J.P. P.O. and N.T. designed the experiments, P.O., V.H.S., J.W., G.F. and A.K. co-wrote the manuscript.

## Additional information

**How to cite this article:** Takada, N. *et al.* A developmental cell-type switch in cortical interneurons leads to a selective defect in cortical oscillations. *Nat. Commun.* 5:5333 doi: 10.1038/ncomms6333 (2014).

## Supplementary Material

Supplementary Figures and Tables Supplementary Figures 1-3 and Supplementary Tables 1-5

## References

[b1] Ascoli G. A. *et al.* Petilla terminology: nomenclature of features of GABAergic interneurons of the cerebral cortex. Nat. Rev. Neurosci. 9, 557–568 (2008). 18568015 10.1038/nrn2402 PMC2868386

[b2] Bartolini G., Ciceri G. & Marin O. Integration of GABAergic interneurons into cortical cell assemblies: lessons from embryos and adults. Neuron 79, 849–864 (2013). 24012001 10.1016/j.neuron.2013.08.014

[b3] Batista-Brito R. & Fishell G. The developmental integration of cortical interneurons into a functional network. Curr. Top. Dev. Biol. 87, 81–118 (2009). 19427517 10.1016/S0070-2153(09)01203-4 PMC4465088

[b4] Fishell G. & Rudy B. Mechanisms of inhibition within the telencephalon: "where the wild things are". Annu. Rev. Neurosci. 34, 535–567 (2011). 21469958 10.1146/annurev-neuro-061010-113717 PMC3556485

[b5] Butt S. J. *et al.* The temporal and spatial origins of cortical interneurons predict their physiological subtype. Neuron 48, 591–604 (2005). 16301176 10.1016/j.neuron.2005.09.034

[b6] Butt S. J. *et al.* The requirement of Nkx2-1 in the temporal specification of cortical interneuron subtypes. Neuron 59, 722–732 (2008). 18786356 10.1016/j.neuron.2008.07.031 PMC2562525

[b7] Takesian A. E. & Hensch T. K. Balancing plasticity/stability across brain development. Prog. Brain Res. 207, 3–34 (2013). 24309249 10.1016/B978-0-444-63327-9.00001-1

[b8] Fagiolini M. & Hensch T. K. Inhibitory threshold for critical-period activation in primary visual cortex. Nature 404, 183–186 (2000). 10724170 10.1038/35004582

[b9] Hensch T. K. *et al.* Local GABA circuit control of experience-dependent plasticity in developing visual cortex. Science 282, 1504–1508 (1998). 9822384 10.1126/science.282.5393.1504 PMC2851625

[b10] Whittington M. A., Traub R. D., Kopell N., Ermentrout B. & Buhl E. H. Inhibition-based rhythms: experimental and mathematical observations on network dynamics. Int. J. Psychophysiol. 38, 315–336 (2000). 11102670 10.1016/s0167-8760(00)00173-2

[b11] Buzsaki G. & Wang X. J. Mechanisms of gamma oscillations. Annu. Rev. Neurosci. 35, 203–225 (2012). 22443509 10.1146/annurev-neuro-062111-150444 PMC4049541

[b12] McBain C. J. & Fisahn A. Interneurons unbound. Nat. Rev. 2, 11–23 (2001). 10.1038/35049047 11253355

[b13] Bartos M., Vida I. & Jonas P. Synaptic mechanisms of synchronized gamma oscillations in inhibitory interneuron networks. Nat. Rev. 8, 45–56 (2007). 10.1038/nrn2044 17180162

[b14] Jonas P., Bischofberger J., Fricker D. & Miles R. Interneuron diversity series: fast in, fast out--temporal and spatial signal processing in hippocampal interneurons. Trends Neurosci. 27, 30–40 (2004). 14698608 10.1016/j.tins.2003.10.010

[b15] Wang X. J. & Buzsaki G. Gamma oscillation by synaptic inhibition in a hippocampal interneuronal network model. J. Neurosci. 16, 6402–6413 (1996). 8815919 10.1523/JNEUROSCI.16-20-06402.1996 PMC6578902

[b16] Whittington M. A., Traub R. D. & Jefferys J. G. Synchronized oscillations in interneuron networks driven by metabotropic glutamate receptor activation. Nature 373, 612–615 (1995). 7854418 10.1038/373612a0

[b17] Tiesinga P. & Sejnowski T. J. Cortical enlightenment: are attentional gamma oscillations driven by ING or PING? Neuron 63, 727–732 (2009). 19778503 10.1016/j.neuron.2009.09.009 PMC2778762

[b18] Sohal V. S., Zhang F., Yizhar O. & Deisseroth K. Parvalbumin neurons and gamma rhythms enhance cortical circuit performance. Nature 459, 698–702 (2009). 19396159 10.1038/nature07991 PMC3969859

[b19] Cardin J. A. *et al.* Driving fast-spiking cells induces gamma rhythm and controls sensory responses. Nature 459, 663–667 (2009). 19396156 10.1038/nature08002 PMC3655711

[b20] Adesnik H. & Scanziani M. Lateral competition for cortical space by layer-specific horizontal circuits. Nature 464, 1155–1160 (2010). 20414303 10.1038/nature08935 PMC2908490

[b21] Atallah B. V., Bruns W., Carandini M. & Scanziani M. Parvalbumin-expressing interneurons linearly transform cortical responses to visual stimuli. Neuron 73, 159–170 (2012). 22243754 10.1016/j.neuron.2011.12.013 PMC3743079

[b22] Lee S. H. *et al.* Activation of specific interneurons improves V1 feature selectivity and visual perception. Nature 488, 379–383 (2012). 22878719 10.1038/nature11312 PMC3422431

[b23] Wilson N. R., Runyan C. A., Wang F. L. & Sur M. Division and subtraction by distinct cortical inhibitory networks in vivo. Nature 488, 343–348 (2012). 22878717 10.1038/nature11347 PMC3653570

[b24] Adesnik H., Bruns W., Taniguchi H., Huang Z. J. & Scanziani M. A neural circuit for spatial summation in visual cortex. Nature 490, 226–231 (2012). 23060193 10.1038/nature11526 PMC3621107

[b25] Nienborg H. *et al.* Contrast dependence and differential contributions from somatostatin- and parvalbumin-expressing neurons to spatial integration in mouse V1. J. Neurosci. 33, 11145–11154 (2013). 23825418 10.1523/JNEUROSCI.5320-12.2013 PMC3718383

[b26] Fox K. A critical period for experience-dependent synaptic plasticity in rat barrel cortex. J. Neurosci. 12, 1826–1838 (1992). 1578273 10.1523/JNEUROSCI.12-05-01826.1992 PMC6575898

[b27] Stern E. A., Maravall M. & Svoboda K. Rapid development and plasticity of layer 2/3 maps in rat barrel cortex in vivo. Neuron 31, 305–315 (2001). 11502260 10.1016/s0896-6273(01)00360-9

[b28] Maffei A., Nelson S. B. & Turrigiano G. G. Selective reconfiguration of layer 4 visual cortical circuitry by visual deprivation. Nat. Neurosci. 7, 1353–1359 (2004). 15543139 10.1038/nn1351

[b29] Turrigiano G. G. & Nelson S. B. Homeostatic plasticity in the developing nervous system. Nat. Rev. Neurosci. 5, 97–107 (2004). 14735113 10.1038/nrn1327

[b30] Brecht M. Barrel cortex and whisker-mediated behaviors. Curr. Opin. Neurobiol. 17, 408–416 (2007). 17702566 10.1016/j.conb.2007.07.008

[b31] Dittgen T. *et al.* Lentivirus-based genetic manipulations of cortical neurons and their optical and electrophysiological monitoring in vivo. Proc. Natl Acad. Sci. USA 101, 18206–18211 (2004). 15608064 10.1073/pnas.0407976101 PMC539748

[b32] Whitten T. A., Hughes A. M., Dickson C. T. & Caplan J. B. A better oscillation detection method robustly extracts EEG rhythms across brain state changes: the human alpha rhythm as a test case. Neuroimage 54, 860–874 (2011). 20807577 10.1016/j.neuroimage.2010.08.064

[b33] Caplan J. B., Madsen J. R., Raghavachari S. & Kahana M. J. Distinct patterns of brain oscillations underlie two basic parameters of human maze learning. J. Neurophysiol. 86, 368–380 (2001). 11431517 10.1152/jn.2001.86.1.368

[b34] Kuhlman S. J. *et al.* A disinhibitory microcircuit initiates critical-period plasticity in the visual cortex. Nature 501, 543–546 (2013). 23975100 10.1038/nature12485 PMC3962838

[b35] Yazaki-Sugiyama Y., Kang S., Cateau H., Fukai T. & Hensch T. K. Bidirectional plasticity in fast-spiking GABA circuits by visual experience. Nature 462, 218–221 (2009). 19907494 10.1038/nature08485

[b36] Liljenstrom H. & Hasselmo M. E. Cholinergic modulation of cortical oscillatory dynamics. J. Neurophysiol. 74, 288–297 (1995). 7472331 10.1152/jn.1995.74.1.288

[b37] Gray C. M. & McCormick D. A. Chattering cells: superficial pyramidal neurons contributing to the generation of synchronous oscillations in the visual cortex. Science 274, 109–113 (1996). 8810245 10.1126/science.274.5284.109

[b38] Buhl E. H., Tamas G. & Fisahn A. Cholinergic activation and tonic excitation induce persistent gamma oscillations in mouse somatosensory cortex in vitro. J. Physiol. 513, (Pt 1): 117–126 (1998). 9782163 10.1111/j.1469-7793.1998.117by.x PMC2231263

[b39] Cunningham M. O., Davies C. H., Buhl E. H., Kopell N. & Whittington M. A. Gamma oscillations induced by kainate receptor activation in the entorhinal cortex in vitro. J. Neurosci. 23, 9761–9769 (2003). 14586003 10.1523/JNEUROSCI.23-30-09761.2003 PMC6740890

[b40] Cunningham M. O. *et al.* A role for fast rhythmic bursting neurons in cortical gamma oscillations in vitro. Proc. Natl Acad. Sci. USA 101, 7152–7157 (2004). 15103017 10.1073/pnas.0402060101 PMC406481

[b41] Rodriguez R., Kallenbach U., Singer W. & Munk M. H. Short- and long-term effects of cholinergic modulation on gamma oscillations and response synchronization in the visual cortex. J. Neurosci. 24, 10369–10378 (2004). 15548651 10.1523/JNEUROSCI.1839-04.2004 PMC6730306

[b42] Cardin J. A., Palmer L. A. & Contreras D. Stimulus-dependent gamma (30-50 Hz) oscillations in simple and complex fast rhythmic bursting cells in primary visual cortex. J. Neurosci. 25, 5339–5350 (2005). 15930382 10.1523/JNEUROSCI.0374-05.2005 PMC3034157

[b43] Pike F. G. *et al.* Distinct frequency preferences of different types of rat hippocampal neurones in response to oscillatory input currents. J. Physiol. 529, (Pt 1): 205–213 (2000). 11080262 10.1111/j.1469-7793.2000.00205.x PMC2270176

[b44] Li X., Morita K., Robinson H. P. & Small M. Control of layer 5 pyramidal cell spiking by oscillatory inhibition in the distal apical dendrites: a computational modeling study. J. Neurophysiol. 109, 2739–2756 (2013). 23486202 10.1152/jn.00397.2012

[b45] Kimura T. *et al.* N3-phenacyluridine, a novel hypnotic compound, interacts with the benzodiazepine receptor. Eur. J. Pharmacol. 311, 265–269 (1996). 8891608 10.1016/0014-2999(96)00434-7

[b46] Takebayashi H., Nabeshima Y., Yoshida S., Chisaka O. & Ikenaka K. The basic helix-loop-helix factor olig2 is essential for the development of motoneuron and oligodendrocyte lineages. Curr. Biol. 12, 1157–1163 (2002). 12121626 10.1016/s0960-9822(02)00926-0

[b47] Novak A., Guo C., Yang W., Nagy A. & Lobe C. G. Z/EG, a double reporter mouse line that expresses enhanced green fluorescent protein upon Cre-mediated excision. Genesis 28, 147–155 (2000). 11105057

[b48] Kusakabe T. *et al.* Thyroid-specific enhancer-binding protein/NKX2.1 is required for the maintenance of ordered architecture and function of the differentiated thyroid. Mol. Endocrinol. 20, 1796–1809 (2006). 16601074 10.1210/me.2005-0327 PMC2588428

[b49] Liguz-Lecznar M. & Skangiel-Kramska J. Vesicular glutamate transporters VGLUT1 and VGLUT2 in the developing mouse barrel cortex. Int. J. Dev. Neurosci. 25, 107–114 (2007). 17289331 10.1016/j.ijdevneu.2006.12.005

[b50] Feldmeyer D., Egger V., Lubke J. & Sakmann B. Reliable synaptic connections between pairs of excitatory layer 4 neurones within a single 'barrel' of developing rat somatosensory cortex. J. Physiol. 521, (Pt 1): 169–190 (1999). 10562343 10.1111/j.1469-7793.1999.00169.x PMC2269646

[b51] Margrie T. W. *et al.* Targeted whole-cell recordings in the mammalian brain in vivo. Neuron 39, 911–918 (2003). 12971892 10.1016/j.neuron.2003.08.012

[b52] Brecht M., Roth A. & Sakmann B. Dynamic receptive fields of reconstructed pyramidal cells in layers 3 and 2 of rat somatosensory barrel cortex. J. Physiol. 553, 243–265 (2003). 12949232 10.1113/jphysiol.2003.044222 PMC2343497

[b53] Kepecs A., Uchida N., Zariwala H. A. & Mainen Z. F. Neural correlates, computation and behavioural impact of decision confidence. Nature 455, 227–231 (2008). 18690210 10.1038/nature07200

[b54] Mitra P. & Bokil H. Observed Brain Dynamics Oxford University Press (2008).

[b55] Brauer K., Hartig W., Bigl V. & Bruckner G. Distribution of parvalbumin-containing neurons and lectin-binding perineuronal nets in the rat basal forebrain. Brain Res. 631, 167–170 (1993). 8298990 10.1016/0006-8993(93)91205-7

