# Supplementary material for: A developmental cell-type switch in cortical interneurons leads to a selective defect in cortical oscillations
Source: Nat Commun. 2014 Oct 30;5:5333. doi: 10.1038/ncomms6333 (PMC4220465; doi:10.1038/ncomms6333)
Supplement: Supplementary Figures and Tables — Supplementary Figures 1-3 and Supplementary Tables 1-5 [file ncomms6333-s1.pdf]

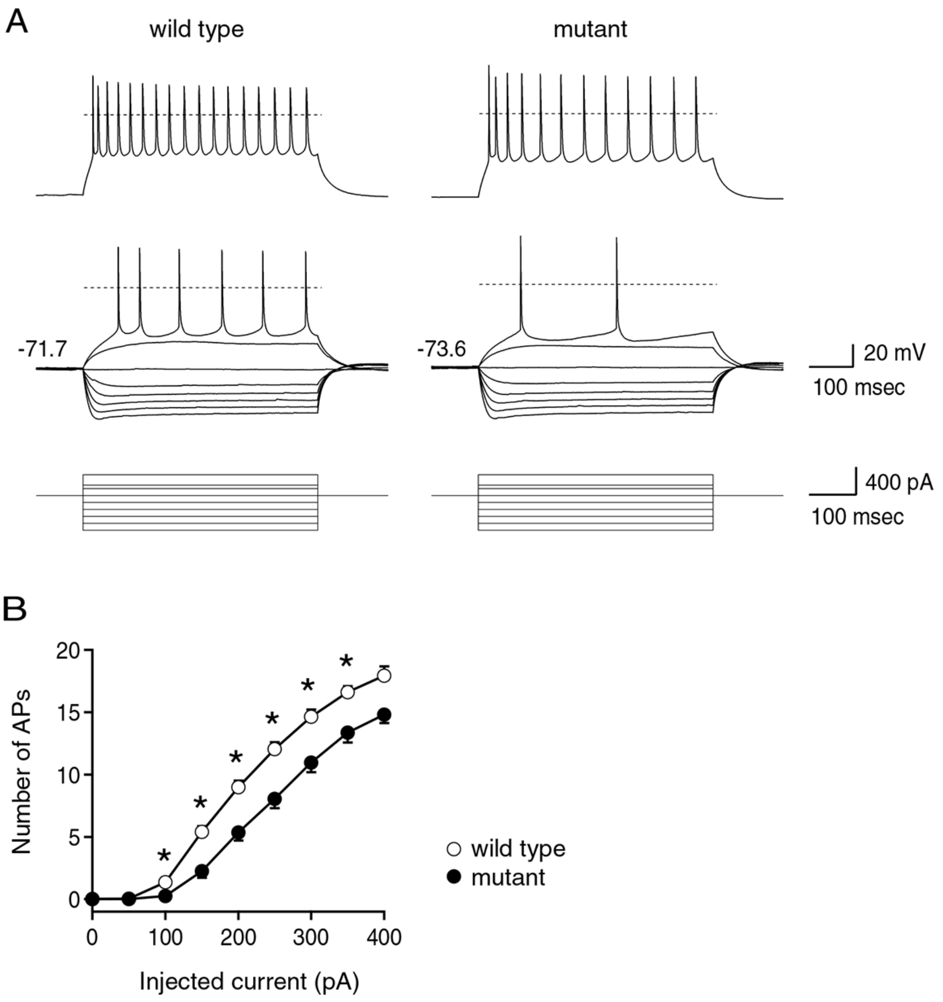

Supplementary Figure 1. Excitability of layer 2/3 pyramidal neurons at P11-13. **(A)** Layer 2/3 pyramidal neurons in control and *Nkx2-1*$^{E12.5LOF}$ slices were subjected to current injections (500 msec DC current from –500 pA, in 50-pA steps; the stimulus traces of each current pulse are shown at the bottom). The mid traces show subthreshold responses and the first suprathreshold response. The upper traces were evoked by an additional 150 pA. The resting membrane potentials (mV) are indicated to the left of the traces (see quantitation of intrinsic and AP properties in Table S1). The dashed lines indicate 0 mV. **(B)** The plot of the number of action potentials evoked by current injections from 0 to 400 pA in 50 pA steps demonstrates lower excitability of layer 2/3 pyramidal neurons recorded in *Nkx2-1*$^{E12.5LOF}$ (black circles) compared to control (white circles) slices. The error bars are SEM; (*) indicates statistical significance, $p < 0.05$. Mann-Whitney *U* test. The corresponding rheobase differences are listed in Table S1.

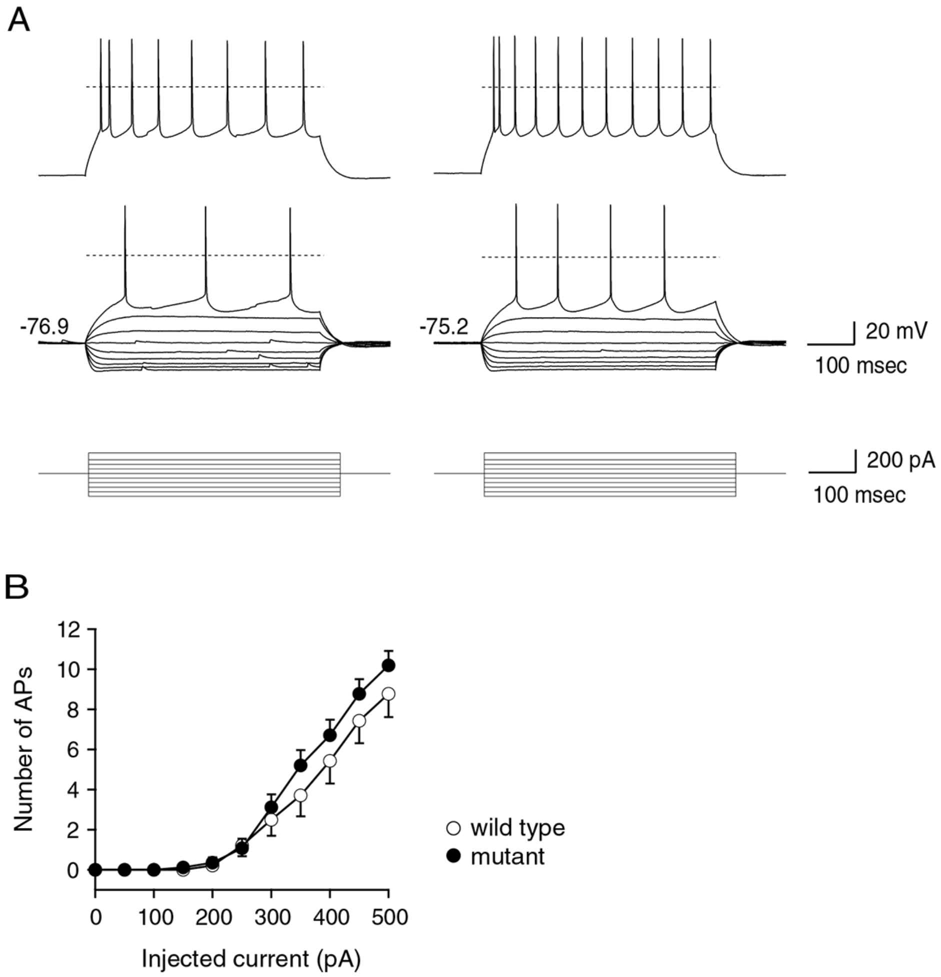

Supplementary Figure 2. Excitability of layer 2/3 pyramidal neurons at P20-25. **(A)** Layer 2/3 pyramidal neurons in control and *Nkx2-1*$^{E12.5LOF}$ slices were subjected to current injections (500 msec DC current from –500 pA, in 50-pA steps; the stimulus traces of each current pulse are shown at the bottom). The mid traces show subthreshold responses and the first suprathreshold response. The upper traces were evoked by an additional 150 pA. The resting membrane potentials (mV) are indicated to the left of the traces (see quantitation of intrinsic and AP properties in Table S2). The dashed lines indicate 0 mV. **(B)** The plot of the number of action potentials evoked by current injections from 0 to 400 pA in 50 pA steps show no statistical differences in excitability between layer 2/3 pyramidal neurons recorded in *Nkx2-1*$^{E12.5LOF}$ (black circles) and control (white circles) slices. The error bars are SEM. (*) indicates statistical significance, $p < 0.05$. Mann-Whitney *U* test. The corresponding rheobase differences are listed in Table S2.

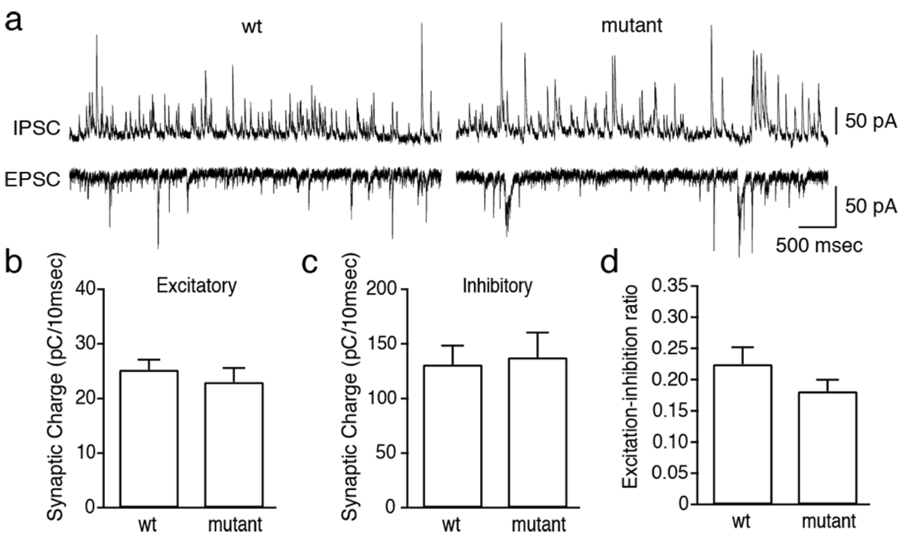

Supplementary Figure 3. Synaptic E/I balance is preserved in the *Nkx2-1*$^{E12.5LOF}$ layer 2/3 cortex. **(A)** Spontaneously evoked IPSCs (top) and EPSCs (bottom) were recorded from barrel cortex layer 2/3 pyramidal neurons in control and *Nkx2-1*$^{E12.5LOF}$ acute brain slices in slightly excitable ACSF (without TTX) in which 2.5 mM KCl and 1 mM $MgCl_2$ were replaced with 4 mM KCl and 0.5 mM $MgCl_2$. **(B)** Analysis of synaptic currents revealed no difference in EPSC (n=12) and IPSC (n=12) synaptic charges between control and *Nkx2-1*$^{E12.5LOF}$ recordings. Error bars are SEM.

	control (cells/mm^2)	Nkx2-1$^{E12.5LOF}$ (cells/mm^2)	p value
superficial PV+	247.6 ± 22.2	17.2 ± 3.1*	<0.0001
superficial SST+	60.0 ± 6.2	8.9 ± 0.4*	<0.001
superficial GABA+	356.6 ± 44.3	279.0 ± 23.1	0.15
superficial WFA+ nests	210.7 ± 12.3	20.2 ± 4.6*	<0.0001
deep PV+	199.7 ± 16.1	148.5 ± 13.5*	<0.05
deep SST+	92.5 ± 13.8	75.4 ± 5.7	0.29
deep GABA+	327.3 ± 37.7	283.1 ± 29.23	0.37
deep WFA+ nests	167.6 ± 6.2	123.8 ± 7.4*	< 0.001

Supplementary Table 1. Cell fate switch of PV+ and SST+ interneurons. Brain slices from 3 to 4 week old *Nkx2-1*$^{E12.5LOF}$ and control littermate mice were immunostained with anti-PV, -SST, -gamma-aminobutyric acid (GABA), and -lectin wisteria floribunda agglutinin (WFA) antibodies; WFA is a marker for visualization of perineuronal nets surrounding PV interneurons (Brauer et al., 1993) (Figure S1). Data are mean ± SEM; (*) indicates statistical significance, $p < 0.05$; pairwise **t**-test; the right column lists the p values for each measurement.

	control	Nkx2-1$^{E12.5LOF}$	p value
Resting membrane potential (mV)	-69.6 ± 0.8	-72.0 ± 0.7*	0.033
Input resistance (MOhm)	149.0 ± 2.7	149.3 ± 4.3	0.95
Rectification index (MOhm/nA)	117.4 ± 4.7	124.9 ± 5.6	0.31
Membrane time constant (msec)	27.3 ± 0.9	26.7 ± 0.8	0.61
Rheobase (pA)	101.8 ± 4.4	127.3 ± 5.9*	0.001
AP threshold (mV)	-36.0 ± 0.4	-37.9 ± 0.4*	0.002
AP amplitude (mV)	75.1 ± 1.0	75.9 ± 1.1	0.603
AP halfwidth (msec)	1.30 ± 0.04	1.48 ± 0.02*	< 0.001
AP rise time (msec)	0.25 ± 0.007	0.29 ± 0.009*	< 0.001
AP decay time (msec)	2.23 ± 0.06	2.43 ± 0.05*	0.015
AHP amplitude (mV)	-15.5 ± 0.3	-13.7 ± 0.5*	0.005
Time to AHP peak (msec)	68.6 ± 2.6	67.3 ± 2.7	0.728

Supplementary Table 2. Biophysical properties in vitro (age P11-13). Recordings were done in brain slices from control (non-induced) littermates and Nkx2-1E12.5LOF mice. Data are mean ± SEM; (*) indicates statistical significance, $p<0.05$; pairwise t-test; the right column lists the p values for each measurement. AHP = afterhyperpolarization.

	control	Nkx2-1$^{E12.5LOF}$	p value
Resting membrane potential (mV)	-77.0 ± 1.0	-73.9 ± 1.0*	0.049
Input resistance (MOhm)	74.4 ± 5.5	73.0 ± 2.7	0.790
Rectification index (MOhm/nA)	80.8 ± 8.4	74.1 ± 4.6	0.449
Membrane time constant (msec)	15.4 ± 0.9	17.0 ± 1.1	0.324
Rheobase (pA)	294.0 ± 23.8	282.3 ± 12.1	0.628
AP threshold (mV)	-35.1 ± 0.7	-34.6 ± 0.8	0.653
AP amplitude (mV)	80.8 ± 0.7	81.2 ± 1.1	0.751
AP halfwidth (msec)	0.94 ± 0.04	0.92 ± 0.02	0.757
AP rise time (msec)	0.23 ± 0.01	0.23 ± 0.01	0.753
AP decay time (msec)	1.54 ± 0.10	1.57 ± 0.06	0.771
AHP amplitude (mV)	-17.8 ± 0.5	-15.2 ± 0.6*	0.012
Time to AHP peak (msec)	45.8 ± 2.4	47.2 ± 1.5	0.602

Supplementary Table 3. Biophysical properties in vitro (age P20-22). Recordings were done in brain slices from control (non-induced) littermates and Nkx2-1E12.5LOF mice. Data are mean ± SEM; (*) indicates statistical significance, $p < 0.05$; pairwise t-test; the right column lists the p values for each measurement.

	control	Nkx2-1$^{E12.5LOF}$	p value
mEPSC (pA)	-16.7 ± 0.9	-20.7 ± 1.4*	<0.05
mIPSCs (pA)	26.0 ± 1.0	34.5 ± 2.2*	<0.05
mEPSC (Hz)	16.5 ± 3.1	15.8 ± 2.1	0.84
mIPSC (Hz)	5.5 ± 0.4	5.2 ± 0.5	0.70
EPSC (pC/10 msec)	25.3 ± 1.8	23.0 ± 2.6	0.48
IPSC (pC/10 msec)	131.1 ± 17.3 8	137.7 ± 22.6	0.82

Supplementary Table 4. Synaptic currents in vitro (age P20-22). Recordings were done in brain slices from control (non-induced) littermates and *Nkx2-1*$^{E12.5LOF}$ mice. Data are mean ± SEM; (*) indicates statistical significance, $p < 0.05$; pairwise **t**-test; the right column lists the p values for each measurement.

	control	Nkx2-1$^{E12.5LOF}$	p value
Resting potential (mV)	-73.5 ± 1.1	-69.4 ± 2.5	0.226
Action potential frequency (Hz)	0.34 ± 0.13	0.08 ± 0.03	0.129
Up-down state □V (mV)	10.8 ± 0.8	8.0 ± 0.7*	0.036
sePSP latency, PW (msec)	9.8 ± 1.5	10.5 ± 2.5	0.841
sePSP latency, SW (msec)	13.3 ± 1.5	12.8 ± 2.0	0.685
sePSP initial slope, PW (mV/msec)	1.0 ± 0.3	1.4 ± 0.7	0.877
sePSP initial slope, SW (mV/msec)	0.27 ± 0.04	0.39 ± 0.08	0.248

Supplementary Table 5. Biophysical and synaptic properties *in vivo*. Recordings were done from anesthetized control (non-induced) littermates and *Nkx2-1*$^{E12.5LOF}$ mice. Data are mean ± SEM; (*) indicates statistical significance, $p < 0.05$; pairwise **t**-test; the right column lists the p values for each measurement.
